# Calibration of Beacons for Indoor Environments based on a Digital Map and Heuristic Information

**DOI:** 10.3390/s19030670

**Published:** 2019-02-06

**Authors:** David Gualda, Jesús Ureña, José Alcalá, Carlos Santos

**Affiliations:** Department of Electronics, University of Alcalá, 28871 Alcalá de Henares, Madrid, Spain; jesus.urena@uah.es (J.U.); jmanuel.alcala@uah.es (J.A.); carlos.santos@uah.es (C.S.)

**Keywords:** beacon calibration, digital map, genetic algorithm, harmonic search, heuristic information, indoor positioning

## Abstract

This paper proposes an algorithm for calibrating the position of beacons which are placed on the ceiling of an indoor environment. In this context, the term calibration is used to estimate the position coordinates of a beacon related to a known reference system in a map. The positions of a set of beacons are used for indoor positioning purposes. The operation of the beacons can be based on different technologies such as radiofrequency (RF), infrared (IR) or ultrasound (US), among others. In this case we are interested in the positions of several beacons that compose an Ultrasonic Local Positioning System (ULPS) placed on different strategic points of the building. The calibration proposal uses several distances from a beacon to the neighbor walls measured by a laser meter. These measured distances, the map of the building in a vector format and other heuristic data (such as the region in which the beacon is located, the approximate orientation of the distance measurements to the walls and the equations in the map coordinate system of the line defining these walls) are the inputs of the proposed algorithm. The output is the best estimation of the position of the beacon. The process is repeated for all the beacons. To find the best estimation of the position of the beacons we have implemented a numerical minimization based on the use of a Genetic Algorithm (GA) and a Harmony Search (HS) methods. The proposal has been validated with simulations and real experiments, obtaining the positions of the beacons and an estimation of the error associated that depends on which walls (and the angle of incidence of the laser) are selected to make the distance measurements.

## 1. Introduction

Indoor Positioning Systems (IPSs) based on the use of an external infrastructure placed in known locations of indoor environments (for instance, on the ceiling of rooms) are an important research topic in the last years. The operation of these systems can be based on different technologies such as radiofrequency (RF) [1,2,3,4], infrared (IR) [5,6], cameras [7,8], ultrasound (US) [9,10] and others. The use of such technologies permits the measurement of distances, or differences of distances, between the tag to be positioned and fixed IPS elements in the infrastructure. These distances feed a positioning algorithm that gives de tag’s position, provided that all the elements in the infrastructure have a known position. 

During the installation phase or in some calibration periods, it is necessary to determine the position of the IPS elements (beacons, infrastructure nodes, cameras, etc.) with respect to the general positioning reference system. This process is known as calibration and, usually, it is carried out manually by using a plumb to determine the projection of the beacons on the floor and solve the 2D position of the beacons from several distances taken from the projected points on the floor to some well-kno wn reference positions of the indoor environment like edges, corners or other known particular marks on the walls. The height of the beacon from the floor is usually obtained by a laser meter. Other method uses an inverse positioning which consists in capturing several distance measures from known test points on the floor to a beacon and, after that, obtaining the position of that beacon using a localization algorithm, in an inverse way, to solve the non-linear equation system [11,12]. The main problem of this method is the need for knowing accurately the positions of a high number of test points on the floor. Other more autonomous methods are based on the use of a mobile robot (MR) with a dead-reckoning system for obtaining the test points from which the distance measurements to the beacon (or to a complete structure of beacons) are made, as in [13]. Nevertheless, when the localization area is large and there are some structures of beacons to cover completely the zone [14], the effort of calibrating all beacons can be quite high, and more in the case in which the position of the beacons can change from time to time. In [10] the solution is the employment of a MR to auto-calibrate sequentially all the structures, but here the problem is that the error related to the position of the beacons is cumulatively increased when the MR moves from a structure of beacons to another (that is, when the MR odometry is used for a long time).

In this paper we propose a calibration of the beacons based on a digital map and some distances easily taken with a laser distance meter. The novelty of this system is the combination of some measurements made to any generic elements of the environment (for instance, the distance to a wall, not to a particular point or landmark on the wall) and heuristic information (like the indication on a map of which wall is and the approximate zone of the map in which the beacons—or their projections on the floor—are) to estimate the position of the beacons. The idea is to take several distance measurements horizontally in two orthogonal directions, using a laser plumb, from the projection of the beacon on the floor to different walls of the environment. The position of a beacon is estimated in a post-processing algorithm based on the use of the map in vector graphic format. In this case, we use a XML format previously developed in [15]. In addition, the heuristic information passed to the algorithm is the estimated region in which the beacons are (it can be roughly drawn on the map), the orientation of the laser plumb (it is only required a coarse value—for example, the orientation of measurements is 90° ± 15°) and the walls to which the distances have been taken.

Our proposal presents some similarities with Simultaneous Localization and Mapping (SLAM) processes [16], in which the approaches are based on the use of a Light Detection and Ranging (LIDAR) device. The aim of these systems is to localize objects or landmarks in the environment and build a map at the same time using the distances measured between the MR and the objects around it (e.g., walls, columns, etc.). A review of the use of different sensors applied to SLAM is reported in [17]. In [18] a LIDAR device is used to determine the indoor 2D location of a mobile robot using a map in AutoCAD (CAD) format. Recently, more complex and advanced approaches include the use of Unmanned Aerial Vehicles (UAVs) in indoor environments to estimate the map and the device´s position in 3D [19,20]. We avoid the use of such systems because we want a system as simpler as possible and are not interested in the map construction (we already have the map of the building), neither in the use of complex measurement systems (such as LIDAR). 

As the problem presented here has not a close solution, we have used two common numerical minimization methods: genetic algorithm (GA) [21] and harmony search (HS) [22,23]. Other optimization methods could be used [24]; note that for us it is more relevant the information included in the calibration algorithm than the particular method used for solving the problem.

The rest of the paper is organized as follows: Section 2 describes the statement of the problem and the information needed to solve it. Section 3 shows some simulation results and the configuration of the optimization methods GA and HS; the proposal is completely validated in Section 4 showing some real experiments; and, finally, some conclusions are presented in Section 5.

## 2. Statement of the Problem

**Figure 1:**
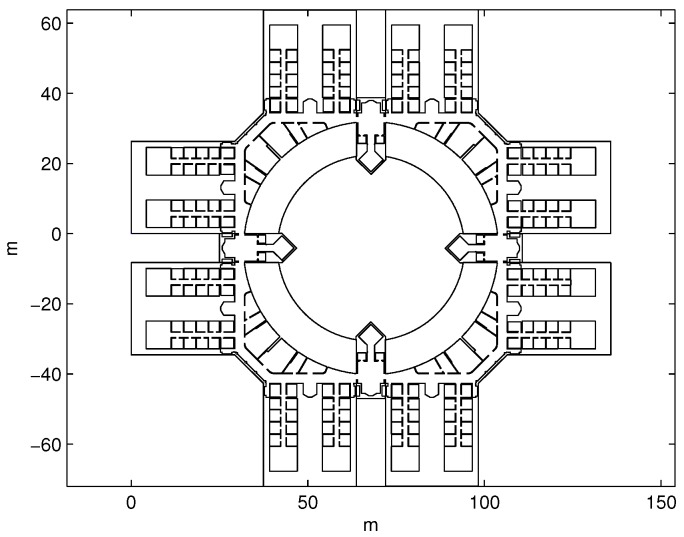
Plan in XML format of the third floor of the Engineering School, University of Alcalá (Spain).

The main requirement to calibrate a beacon using the proposal of this paper is to have a map of the environment in a vector graphic format where the beacons are going to be placed. For testing our proposal we have used a floor plan in XML format developed in [15]. A part of this map in which the simulations and experiments were carried out is shown in Figure 1 and corresponds to the third floor of the Engineering School, University of Alcalá (Spain).

**Figure 2:**
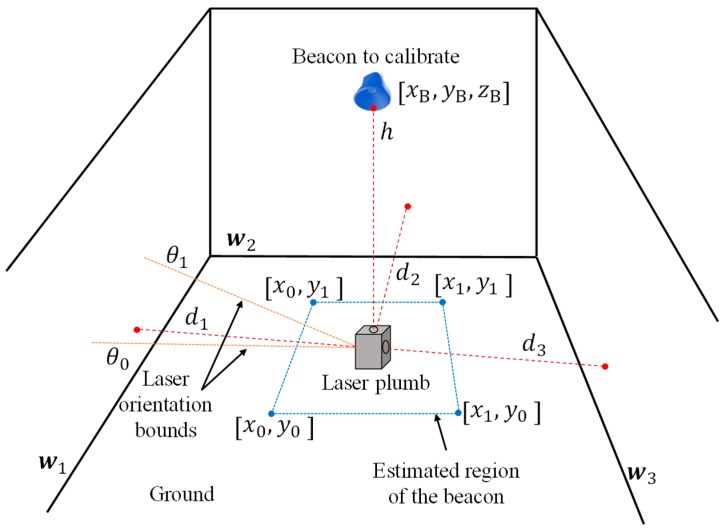
General diagram for the statement of the problem.

To estimate the position of a beacon, as have been pointed out, it is necessary to collect these input data: several distances from the beacon’s projection on the floor to the walls and some heuristic information, such as the approximate region in which this beacon’s projection is and the bounds of the laser structure orientation. Figure 2 shows a 3D diagram in which these data inputs are represented. 

The laser plumb is placed on the ground in the position where the beacon on the ceiling to be calibrated is pointed by the laser ray emitted from the top of the laser plumb. In addition to this ray, the laser plumb emits other three rays in the horizontal plan (always in directions of 90° of separation one each other). The distances from the laser plumb to the walls in the points marked by these rays are measured. In the representation of Figure 2, there are three horizontal ray projections since it is based on the real laser plumb used after in the experiments. Note that the impact of two rays could be on the same wall, which is a typical case of long corridors. In general, the number of distances from the laser plumb to the walls can be N, as expression (1) shows:

(1) $$D=\left[\begin{array}{c} d_{1}\\ \vdots \\ d_{n}\\ \vdots \\ d_{N} \end{array}\right]$$

The heuristic information related to the bounds of the estimated region of the X-Y beacon’s position, the approximate orientation of the laser plumb and the walls on which each laser projection impacts are detailed in the next paragraphs.

The approximate rectangular X-Y region in which the beacon is placed is defined by the vector R as:

(2) $$R={\left[\begin{array}{cccc} x_{0} & x_{1} & y_{0} & y_{1} \end{array}\right]}^{T}$$

This region can be selected as large as the user estimates to be sure the beacon’s projection is inside it. The vector that represents the bounds of the angle related to the laser plumb orientation is defined as:

(3) $$A={\left[\begin{array}{cc} \theta_{0} & \theta_{1} \end{array}\right]}^{T}$$

In the same way as for the region, these values must be selected by the user large enough to guarantee they include the real orientation of the laser plumb. 

At last, W in expression (4) represents the identification of the walls (from *w*_1_ to *w_N_*) in the vector map where each horizontal laser impacts, also to be used by the minimization algorithm:

(4) $$W=\{\begin{array}{c} w_{1}\\ \vdots \\ w_{n}\\ \vdots \\ w_{N} \end{array}$$

All inputs explained before are included in the algorithm that obtains the estimation of the position of the beacon $\hat{B}$ (X-Y coordinates and the orientation, θ, that can be or interest in some cases) according to the minimization of the fitness function f, given in (6), using the measured distances and the constraints provided by the heuristic information:

(5) $$\hat{B}={\left[\begin{array}{ccc} {\hat{x}}_{B} & {\hat{y}}_{B} & \hat{\theta } \end{array}\right]}^{T}$$

(6) $$f=\frac{1}{N}\cdot \overset{i=N}{\underset{i=1}{\sum }}\left|d_{i}-{\hat{d}}_{i}\right|$$

where $d_{i}$ is the $i^{th}$ measured distance from the projection of the beacon on the floor to each wall, and ${\hat{d}}_{i}$ is the $i^{th}$ estimation of such distance using the estimated position of the beacon $\hat{B}$ given by the optimization algorithm for each iteration (for the first iteration this distance is calculated from a random point in the search region).

As said before, we use two popular numerical solvers: genetic algorithm (GA) [21] and harmony search (HS) [22] for this approach of combining mapping information and heuristic data to calibrate the sensors with the lowest effort as possible.

**Figure 3:**
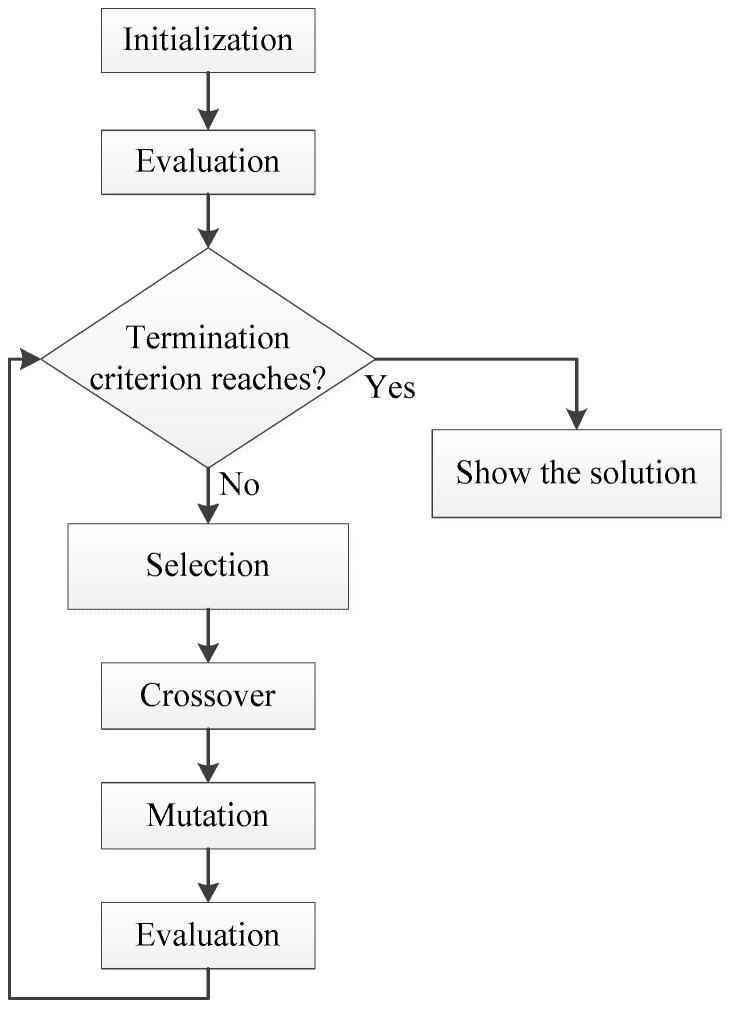
Genetic Algorithm flowchart of the basic steps.

A basic scheme which contains the original steps of the GA is shown in Figure 3. This algorithm gives a solution of an optimization problem using an imitation of the natural selection process, in which several genetic events such as mutation or crossover are used for solving the problem.

The initialization step consists of setting the parameters of the algorithm and creating the first generation of chromosomes (possible solutions to the optimization problem). Then, in the evaluation process the chromosomes are scored to determine the best solutions of such generation. If the termination criterion is satisfied, the algorithm finishes showing the best chromosome (solution); if not, the next step is the selection of the chromosomes for generating the next set of new chromosomes. Each of them has a probability of being selected equal to its score divided by the total scores of the generation. The following step is to apply several genetic operators (crossover and mutation) to create the new population of solutions. In the crossover phase, the chromosomes are paired up with a particular probability of crossing. After the crossover, several genes of the chromosome are mutated according to a probability. Then, the new generation of chromosomes is evaluated and the termination criterion is checked again. The generation of new chromosomes continues with the steps described before until a termination criterion is satisfied, and the algorithm will show the best solution of the optimization problem.

**Figure 4:**
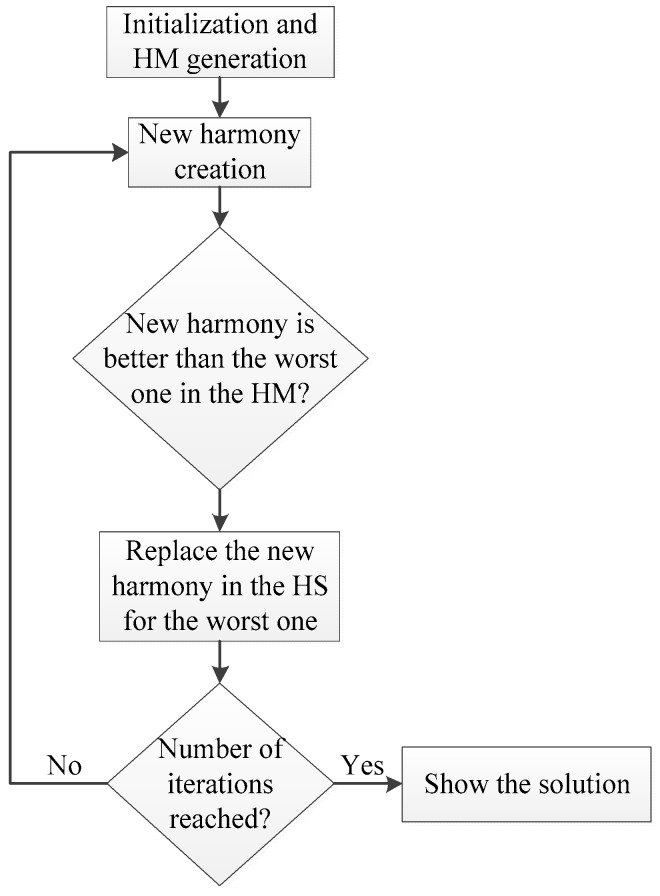
Harmony Search flowchart of the basic steps.

In the harmony search (HS) method, the improvisation process of a musicians’ band is used as the base for solving optimization problems, so the goal is to find the perfect state of harmony. The objective function is represented by the audience´s aesthetics; the decision variables are represented by each musician; the pitch range of a musical instrument is analogous to the value range of the decision variables and the musical harmony at a specific time is equivalent to a solution vector of a single iteration. The set of solutions vector is saved on a harmony memory (HM) with a previous configured size and it is the equivalent of the population in the GA. Figure 4 shows a summarized flowchart of this algorithm.

Once the initialization parameters are configured, the first generation of harmonies (possible solutions of the problem) are generated and saved on the HM according to the size previously set (HMS). Then, a new harmony is created based on several parameters: the harmony memory considering rate (HMCR) with a range from 0 to 1, controls the influence of the harmonies in the HM to generate the new one.

For example, if HMCR is equal to 0, the new harmony is completely random; the pitching adjust rate (PAR) determines the probability of a harmony from the HM to be mutated; and the bandwidth (BW) is the limit change in the pitch adjustment. Then, if the new harmony is better than the worst harmony in the HM, the new one is replaced by the worst one in the memory. Finally, if the number of iterations is reached, the algorithm provides the best solution of the HM; if not, a new harmony is generated again according to the parameters previously described and the evaluation process is repeated. 

## 3. Simulated Results

Using a Graphical User Interface (GUI) developed in MatLab we can set a beacon or sensor around the localization area and emulate the behavior of the laser plumb projections including the information of the distances from the projected beacon on the ground to three projected walls provided by the laser meter or another distance meter.

**Figure 5:**
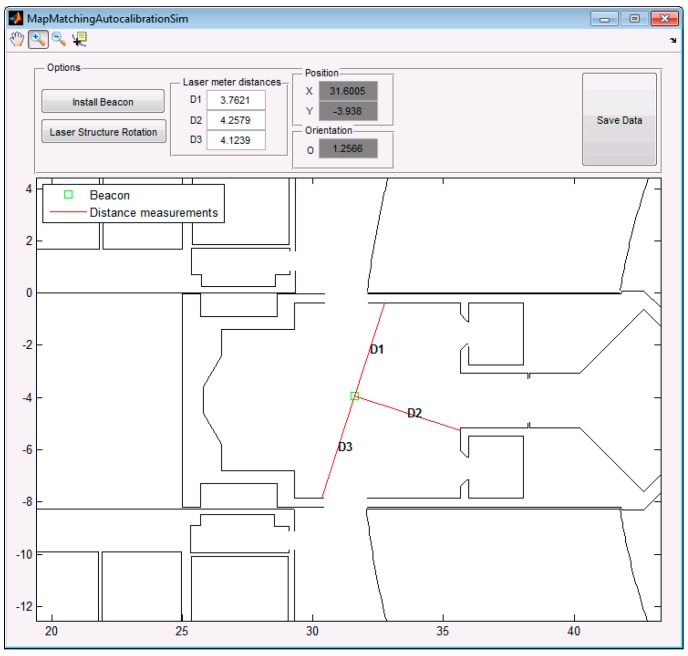
MatLab GUI that emulates the calibration system.

Figure 5 shows the 2D interface of this emulator with a beacon placed at an arbitrary position and the three distance measurements of the laser plumb projections for each sensor including different orientations of the laser structure.

As can be seen, the panel “Position” shows the real coordinates in meters of the beacon, and the panel “Orientation” the angle in radians of the laser plumb (direction in which D1 was taken regarding the x axis). The ground truth of the three distances is represented in the panel “Laser meter distances”. These distances will be contaminated in the simulation with a Gaussian noise in a high number of trials in order to evaluate the performance of the algorithm using GA and HS methods for estimating the position of the sensor.

The common problem related to these meta-heuristic optimization algorithms is the configuration of the parameters such as the set size of solutions or the number of iterations to provide the results. The recognized reference [23] recommends that an appropriate size of the set of solutions is equal to 10 times the dimension of the optimization problem. In this case there are three variables to be estimated $\left({\hat{x}}_{B},\;{\hat{y}}_{B},\;\hat{\theta }\right)$, so the set size of solutions is 30.

Regarding the number of generations or iterations of GA and HS we have realized a study to determine an appropriate number of iterations to solve the proposed optimization problem. We have evaluated several numbers of iterations according to the size of the heuristic information related to the approximate rectangle where the beacon is placed and the bounds of the laser plumb orientation. For example, if the approximate region is a square of $9\;m^{2}$ and the increment of the orientation bounds related to the laser structure is $\frac{\pi }{4}rad$ there are around 7,020,000 combinations to find the position of the beacon and the laser plumb orientation that minimizes the fitness function according to the three distances from the projection of the beacon to each wall with two decimals of precision. Therefore, the idea is to evaluate different numbers of the total combinations using GA and HS to determine the appropriate number of iterations according to a percentage of all possible combinations. We have used the values by default related to the rest of parameters of the GA and HS algorithms, and the most important are summarized in Table 1 and Table 2, respectively.

We have realized the study to determine an appropriate number of generations or maximum iterations using the data of the example previously showed in Figure 5, in order to use the appropriate value in the rest of simulations and the experimental results. We have estimated the position of the beacon 100 times for each number of generation or iterations, and the range of that number is from 0.01% to 0.2% of the amount of possible combinations assuming a precision with two decimals.

**Figure 6:**
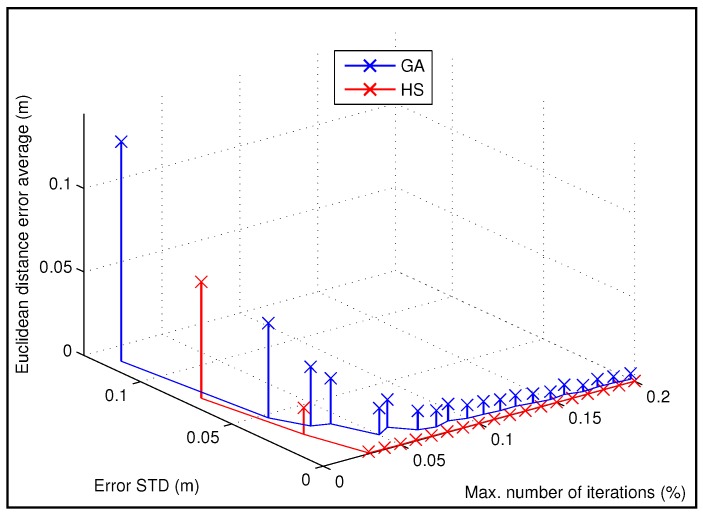
Study of the error based on the Euclidean distance and the standard deviation (STD) for the calibration of a beacon according to several numbers of iterations represented by a percentage of possible combinations to solve the problem, comparing GA and HS methods.

Figure 6 presents the simulated results according to the study of the number of iterations needed (in percentage over all possible combinations), also showing the mean and standard deviation per number of generations (iterations), comparing the GA and HS algorithms. In addition, the results are detailed in Table 3.

According to the Figure 6 and Table 3, the performance using the HS method is much better than the GA. For 0.03% of iterations (in percentage over all possible combinations), the mean of the error in the estimation of the position of the beacon is 4 cm for GA and lower than 1 mm for HS. From 0.05% to the end, the mean error for HS optimization is stabilized at a value lower than 0.5 mm and the minimum average error achieved by GA is lower than 0.5 cm from 0.15% to the end of iterations considered. 

For the rest of simulations and experimental tests we have selected a maximum number of iterations or generations equal to the 0.1% over all the possible combinations, according to the previous study. Note that for that value the average error is stabilized at a value lower than 1cm for the GA and the error for the same number of iterations using the HS algorithm is lower than 0.5 mm. In addition, the execution time for 0.1% of iterations in the previous example is around 30 s. It is worth noting that for this application the computing time is not a critic constraint as the calibration process is a task that is necessary to be realized only reduced number of times (the first time the beacon is installed and after some reparation or change). 

**Figure 7:**
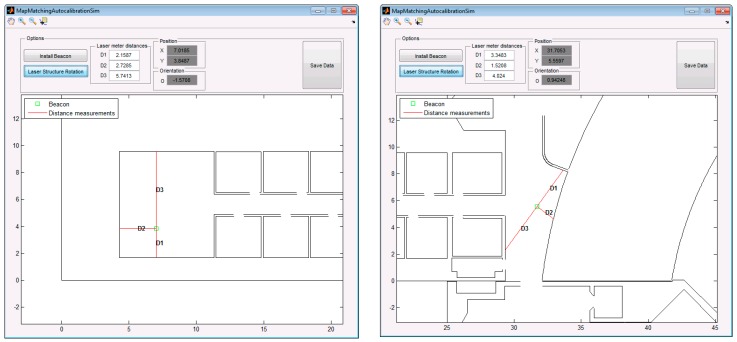
Simulated calibration of two beacons: beacon #1 (left) and beacon #2 (right).

Figure 7 shows the position of two beacons to be calibrated using the simulation interface and the corresponding information related to the distances and the ground truth. For estimating the position of each beacon and evaluating the behavior of the algorithms we have realized 100 estimations per beacon adding a Gaussian noise in the distance measurements based on real characteristics of a laser meter (e.g., 2 mm of accuracy in the 95% of cases [25]). In this case we have also used the same heuristic information than in the previous study: an approximate X-Y region of $9\;m^{2}$ and the orientation bounds related to the orientation of the laser plumb equal to $\frac{\pi }{4}rad$.

**Figure 8:**
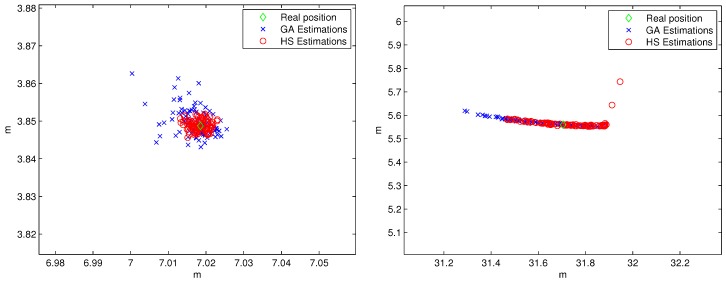
Simulated results for the estimations of beacon #1 (left) and beacon #2 (right).

Figure 8 shows the visual results of the 100 estimated points for the calibration of each beacon using GA and HS. Note that the X-Y scales are completely different in these two subplots.

It can be appreciated that the maximum error for the estimation of beacon #1 is around 2 cm and lower than 1 cm for GA and HS method, respectively. Nevertheless, in the case of the estimations of beacon #2 the errors using both methods increase a lot compared to the first case, being the errors in the decimeter order. It is due to the combination of the distance measurement errors with the shape of the walls selected to estimate the position, since in this case the first distance is measured using a wall with a high inclination and the shape of the second one is a curve. 

**Figure 9:**
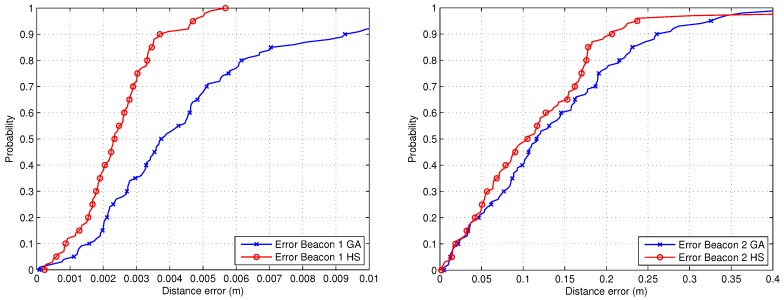
CDF of the error related to the estimation of the positions of beacon #1 (left) and beacon #2 (right).

Finally, Figure 9 presents a cumulative distribute function (CDF) of the error in both calibrations, showing that the performance of the HS optimization method is much better than the GA, achieving errors lower than 3.5 mm in the 80% of cases for beacon #1 using the HS algorithm, in comparison with an error in the same situation less than 6.5 mm for the GA method. 

Regarding the error for the calibration of beacon #2, in the 80% of cases it is lower than 18 cm for the HS method and less than 23 cm for the GA approach. Note that the second calibration was carried out using an adverse selection of the walls to measure the distances from the projection of the beacon, since the shape of some of these walls are very inclined (regarding the direction of measurement) and in one case curved. 

In order to see the effect of the number of distance measures considered, we have performed a simulation with two, three and four distances. Note that other situations, with more measurements could be interesting for SLAM with a laser scan, but this is not our case as we are interested in a calibration as simpler as possible. Table 4 details the numeric results related to the errors of the position estimation using different number of distances measured. 

Note that the case of four distances cannot be used in our real experiments since the laser plumb used provides only three horizontal projections. The trend is that the error is decreased according to the use of more distances. The most significant reduction occurs when the number of distances is increased from two to three. This is due to the high number of cases in which the result of the fitness function (Equation (6)) is near to the minimum (there is a high uncertainty with only two measures).

The Euclidean distance error average for the estimation of beacon #1 is established when the number of distances is three, since the difference in the error adding one more distance is lower than 1 mm. For the beacon #2 position estimation, there is a high difference if the number of distances is increased from three to four due to the bad geometry selected (high inclination of the walls, curved walls, etc.). Therefore, the recommendation for the user is that the best approach for calibrating the beacons with a minimum error is to select straight walls with approximately perpendicular measurements to them with the laser plumb.

Another important point is the distance meter accuracy. In this case we have supposed a standard deviation of 2 mm in the distance measuring uncertainty based on a real laser meter features. Nevertheless, if the quality of the distance meter is worse (e.g., an ultrasonic distance meter), the calibration error of the beacon increases. Table 5 shows the results supposing that the standard deviation of the distance meter is 5 mm and 1 cm. 

The results show that the effect of this increment in the standard deviation of the distance measuring uncertainty is not a critical factor in the calibration of the beacons, obtaining a maximum average distance error of around 1 cm for the estimation of beacon #1 using the GA and HS methods, 0.1582 m for beacon #2 using the GA method, and 0.1396 m for HS algorithm, representing a low increment of the error with respect to the initial study in which the standard deviation of the distance measures noise was 2 mm.

We have also analyzed a possible skewness effect in the distance measuring noise, considering a skew factor of 25% and 50% related to the standard deviation of the measurement uncertainty (maintaining the mean to zero). Table 6 shows the results in terms of the Euclidian distance error average in calibration according to the skewness effect, which is obtained with the Pearson system random numbers in MatLab.

It can be observed that adding a skewness factor in the distance measuring noise, in which the mode value is displaced from the zero-mean and the STD is 2 mm, the results related to the calibration of beacon #1 are consistent and there is a low increment of the Euclidean distance error average in the estimation of the beacon #2 position, due to the worse geometry conditions of the measurements. Nevertheless, it is a fact that if the measures are biased, the precision of the estimation of the beacons would be the same, but not the accuracy (trueness), which would be worsen.

Since the skewness factor only affects the mode of the distance measurement errors, we have carried out the last simulations supposing that the measures are biased. Table 7 shows the results according to the addition of two possible offsets (2 mm and 5 mm) to the distance measures.

In this case, the Euclidean distance error average is increased according the increment of the bias, so the accuracy of the system worsens, but not the precision that is kept approximately constant. Note that the increase in error is not too high (until millimeters) because the bias is also around millimeters and the distance measurements can be of several meters.

## 4. Experimental Results

**Figure 10:**
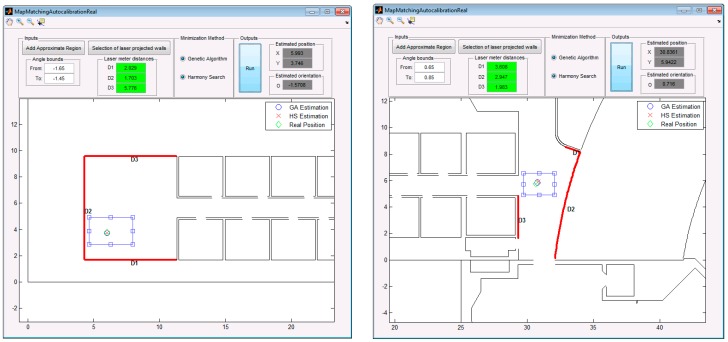
Real data included in the developed GUI related to a single position estimation for two placements of beacons (beacon #1 on the left and beacon #2 on the right) in two different environments (same as those used for simulation in Figure 7).

In order to validate the proposal of this paper and the simulations of the previous section, we have carried out experimental tests in similar locations and type of walls than those used for the simulation experiments (see Figure 10). 

The MatLab GUI used in simulations has been adapted to introduce the information of the three distances to walls and the heuristic data needed by the algorithm (approximate rectangular region, the walls on the map and the angle bounds for the orientation of the laser plumb). In addition, it is possible to select the method for estimating the position of the beacon (GA or HS).

**Figure 11:**
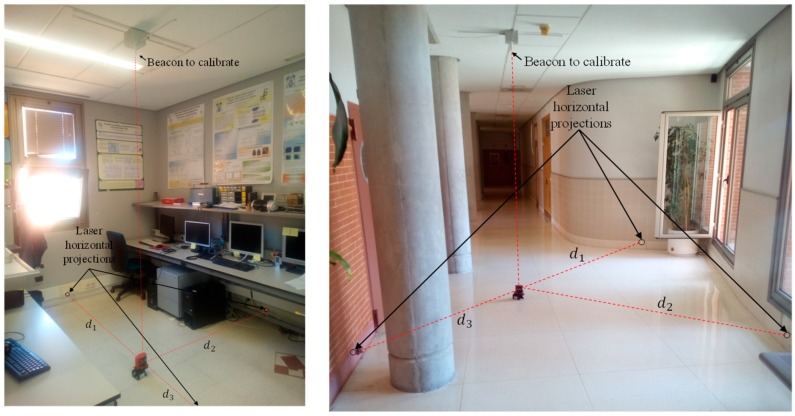
Experimental calibration of two ULPSs placed on similar locations than those used for the simulated results. Beacon #1 (left) placed on the ceiling of a laboratory and beacon #2 (right) located on the ceiling of a corridor.

Figure 10 presents the interface used for calibrating the beacons in the real environment, with real measurements. A picture of the real environments can be seen in Figure 11, in which it is shown the process of calibration of the central beacon of an ultrasonic local positioning system (ULPS) in the two situations considered. The rest of the LPS beacons are estimated from this one since all the LPS has a predetermined and known structure (note that the “orientation” of the central beacon has sense in this context –it is considered regarding the other beacons).

To characterize the system using a CDF, we have repeated the experiment 10 times for each location, with a little and random rotation of the laser plumb before each trial. We propose the rotation of the laser plumb instead of turning off and on the laser meter due to the high precision of the measurement system (without changes in the reading of the laser distance measure in most of iterations and 1 mm of change in a few attempts). The real position (ground truth) of the central beacon for all cases has been obtained manually using a measuring tape, for obtaining the positioning errors. 

**Figure 12:**
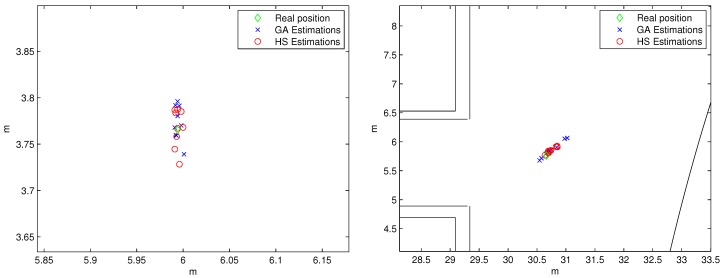
Experimental results for the estimations of beacon #1 (left) and beacon #2 (right). Note that the X-Y scales are completely different in these two subplots.

Figure 12 shows a cloud of points of the results of the estimations in both environments using GA and HS algorithms. In this case, the maximum error in the estimation of the beacon #1 for both methods (GA and HS) is in the order of centimeters (some millimeters for the first time, in which the laser projections are nearly perpendicular, so it is the most favorable situation); whereas it grows up to decimeters for the second scenario (beacon #2), being 0.13 m and 0.12 m the error related to the first measures, for the GA and HS methods, respectively. The HS method for the position estimation of beacon #2 achieved better results than the GA, since their estimations present lower dispersion around the real position. In the estimation of the beacon #1 position, the performance of HS and GA algorithms is similar, mainly due to the fact that the number of iterations for solving this case is enough for both cases. Nevertheless, it is a fact that HS algorithm needs less iterations than GA to offer an appropriate solution of the position estimation. If the computing time is not a constraint (for instance, if the calibration is made only in the installation phase or occasionally after), the number of iterations of the GA method could be increased to have similar performances. 

**Figure 13:**
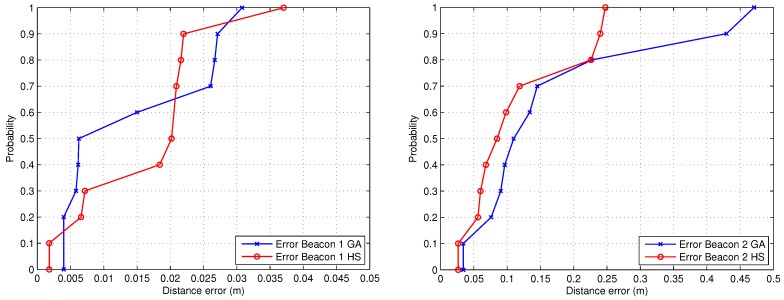
CDFs of the error related to the estimation of the positions for beacon #1 (left) and beacon #2 (right).

Finally, Figure 13 shows the CDF representation of the experimental results. The positioning errors are lower than 3 cm for beacon #1 (90% of the cases) using both methods, and lower than 25 cm (90% if the cases for HS) in the case of beacon #2, bearing in mind the unfavorable measurement conditions. Note that a calibration error in the range of centimeters can be suitable for the majority of applications related to indoor positioning (people and mobile robot navigation).

**Figure 14:**
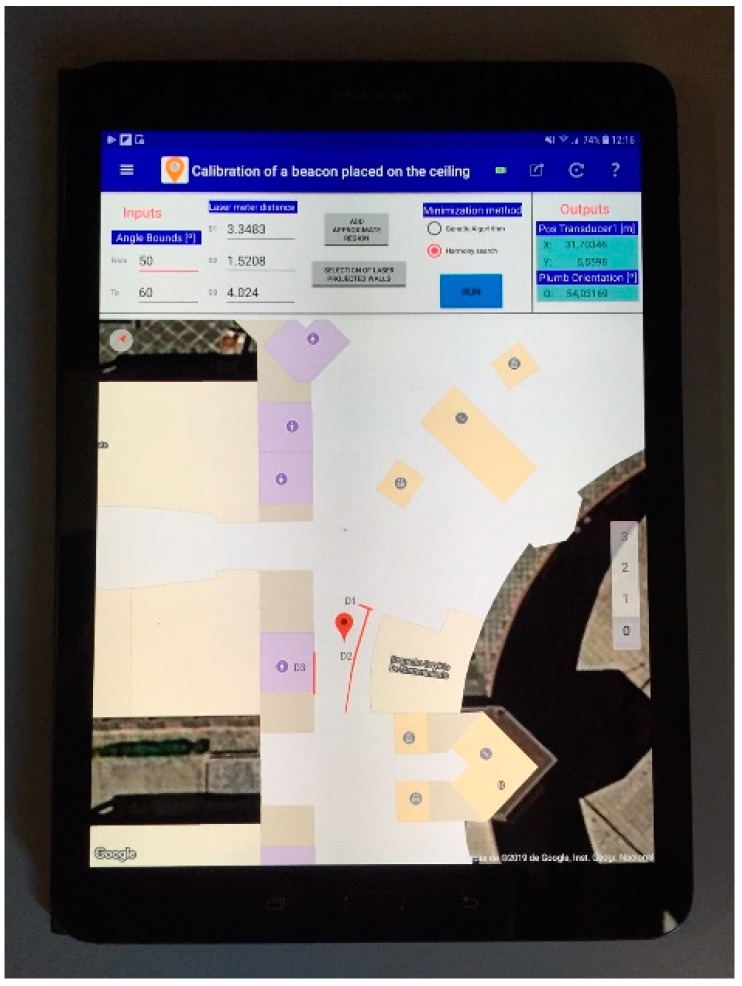
Android version of the proposal running on a tablet.

Figure 14 shows the first version of the proposal running in an Android device, using the Google indoor map of the building.

## 5. Conclusions

In this paper we have carried out a proposal for calibrating the position of beacons placed on the ceiling that compose an indoor localization system, mainly using the map information in vector graphic format. The proposal consists of taking three measurements from the beacon’s projection on the floor to three neighbor walls using a laser plumb. This information is the input of an optimization algorithm in addition to other heuristic data, such as the approximate XY area in which the beacon is placed, an indication on the map which the three walls used and the approximate bounds of the laser plumb orientation. 

The algorithm estimates the position of the beacon based on the genetic algorithm (GA) and harmony search (HS) meta-heuristic methods. Simulation results show that HS optimization needs less iterations than GA to obtain an appropriate solution with similar precision. The calibration errors obtained range from the order of few centimeters when the measurements are taken in a typical case with appropriate conditions (laser plumb in perpendicular with respect to straight walls) and in the order of decimeters when the conditions are quite unfavorable (curve and diagonal walls with oblique measurements with the laser plumb). 

The proposal has been validated with experimental results, in similar environment conditions than those used for simulations. The calibration errors are lower than 3 cm in the 80% of cases in the case that the measurements were carried out with straight walls and perpendicular orientations of the laser plumb, and lower than 25 cm in the 80% of cases in the worst analyzed situation. Therefore, these results allow calibrating beacons in indoor environments when the map is available in a vector graphic format with centimetric precision and a considerable reduction of the man-effort compared to the usual manual process. 

It is possible to obtain the position of the beacons as it is obtained in the ground truth by a measuring tape, but the process requires much more effort: at first, we have to measure several distances manually (with the measuring tape) from the projection of the beacon on the floor to different known points of the environment (e.g., corners) and it has to be accurate, so it is necessary to analyze the map in order to extract the coordinates of that corners; then, we have to apply an equation system to solve for the position (note that the map is needed anyway). With the proposal described in this paper, we measure the three projected points of the laser plumb with a laser meter, and using the GUI developed in MatLab or an Android app we introduce these distances, the selection of the three walls (clicking on the screen), an approximate region in which we guess the beacon is and the approximate bounds of the laser plumb orientation. All these inputs are fast to enter in the interface and without the proposal we have to analyze the map and extract several reference points. Finally, we press a button to obtain the estimation of the beacon position. Depending on the part of the environment the difference between doing the process manually or using the GUI could be a long time. Using the app it is easy to spend no more than a couple of minutes to get the position, including the measuring stage, since it is not necessary to exactly measure a specific point of the map (as occurs in the manual process with the corners or other landmarks). 

## Figures and Tables

**Table 1:** Several configuration parameters of the genetic algorithm.

Meta-Heuristic Method	Population Size	Generations	Crossover Fraction	Time Limit
Genetic Algorithm	30	From 0.01% to 0.2% of total combinations assuming a precision with 2 decimals.	0.8	∞

**Table 2:** Several configuration parameters of the harmony search method.

Meta-Heuristic Method	Harmony Memory Size (HMS)	Maximum Number of Iterations	Harmony Memory Consideration Rate (HMCR)	Pitch Adjusting Rate (PAR) Min/Max	Bandwich (BW) Min/Max
Harmony Search	30	From 0.01% to 0.2% of total combinations assuming a precision with 2 decimals.	0.9	0.1/0.5	0.01/0.05

**Table 3:** Detailed results of the study of the maximum number of iterations needed for GA and HS methods.

Max. Number of Iterations (%)	Euclidean Distance Error Average (m) (GA)	Euclidean Distance Error Average (m) (HS)	STD of the Error (m) (GA)	STD of the Error (m) (HS)
0.01	0.13	0.07	0.12	0.07
0.02	0.05	0.02	0.05	0.03
0.03	0.04	<0.001	0.03	<0.001
0.04	0.03	<0.001	0.03	<0.001
0.05	0.02	<0.0005	0.01	<0.0005
0.06	0.02	<0.0005	0.01	<0.0005
0.07	0.01	<0.0005	<0.01	<0.0005
0.08–0.14	<0.01	<0.0005	<0.01	<0.0005
0.15–0.20	<0.005	<0.0005	<0.005	<0.0005

**Table 4:** Results according to the number of distances used for estimating the position of the beacon.

Method	Beacon	Two Distances		Three Distances		Four Distances	
		Euclidean Distance Error Average (m)	STD of the Error (m)	Euclidean Distance Error Average (m)	STD of the Error (m)	Euclidean Distance Error Average (m)	STD of the Error (m)
**GA**	Beacon #1	0.1515	0.1701	0.0048	0.0036	0.004	0.0023
	Beacon #2	0.8173	0.5843	0.1380	0.0965	0.0088	0.0059
**HS**	Beacon #1	0.0815	0.0785	0.0024	0.0012	0.0022	0.0012
	Beacon #2	0.5044	0.3353	0.1245	0.1102	0.0025	0.0014

**Table 5:** Results according to different values of the standard deviation related to the distance measuring noise.

Method	Beacon	STD of 2 mm		STD of 5 mm		STD of 1 cm	
		Euclidean Distance Error Average (m)	STD of the Error (m)	Euclidean Distance Error Average (m)	STD of the Error (m)	Euclidean Distance Error Average (m)	STD of the Error (m)
**GA**	Beacon #1	0.0048	0.0036	0.0075	0.0041	0.0111	0.0063
	Beacon #2	0.1380	0.0965	0.1536	0.1090	0.1582	0.1119
**HS**	Beacon #1	0.0024	0.0012	0.0055	0.0028	0.0114	0.0061
	Beacon #2	0.1245	0.1102	0.1324	0.1045	0.1396	0.1002

**Table 6:** Results adding a skewness factor in the distance measuring noise.

Method	Beacon	Without Skewness		25% of Skewness with Respect to the Standard Deviation of 2 mm		50% of Skewness with Respect to the Standard Deviation of 2 mm	
		Euclidean Distance Error Average (m)	STD of the Error (m)	Euclidean Distance Error Average (m)	STD of the Error (m)	Euclidean Distance Error Average (m)	STD of the Error (m)
**GA**	Beacon #1	0.0048	0.0036	0.0047	0.0038	0.0049	0.0033
	Beacon #2	0.1380	0.0965	0.1498	0.1090	0.1597	0.1175
**HS**	Beacon #1	0.0024	0.0012	0.0023	0.0013	0.0024	0.0012
	Beacon #2	0.1245	0.1102	0.1326	0.1215	0.1373	0.1222

**Table 7:** Results adding several biases in the distances measured, with a standard deviation equal to 2 mm.

Method	Beacon	Without Bias		2 mm of Bias		5 mm of Bias	
		Euclidean Distance Error Average (m)	STD of the Error (m)	Euclidean Distance Error Average (m)	STD of the Error (m)	Euclidean Distance Error Average (m)	STD of the Error (m)
**GA**	Beacon #1	0.0048	0.0036	0.0051	0.0035	0.0053	0.0032
	Beacon #2	0.1380	0.0965	0.1419	0.1095	0.1574	0.0981
**HS**	Beacon #1	0.0024	0.0012	0.0025	0.0014	0.0037	0.0014
	Beacon #2	0.1245	0.1102	0.1264	0.1059	0.1266	0.1081
